# Strong Suppression of Thermal Conductivity in the Presence of Long Terminal Alkyl Chains in Low‐Disorder Molecular Semiconductors

**DOI:** 10.1002/adma.202008708

**Published:** 2021-08-03

**Authors:** Ekaterina Selezneva, Alexandre Vercouter, Guillaume Schweicher, Vincent Lemaur, Katharina Broch, Aleandro Antidormi, Kazuo Takimiya, Veaceslav Coropceanu, Jean‐Luc Brédas, Claudio Melis, Jérôme Cornil, Henning Sirringhaus

**Affiliations:** ^1^ Optoelectronics Group Cavendish Laboratory University of Cambridge JJ Thomson Avenue Cambridge CB3 0HE UK; ^2^ Laboratory for Chemistry of Novel Materials University of Mons Mons 7000 Belgium; ^3^ Institut für Angewandte Physik Universität Tübingen Auf der Morgenstelle 10 72076 Tübingen Germany; ^4^ Catalan Institute of Nanoscience and Nanotechnology (ICN2) CSIC and BIST Campus UAB, Bellaterra Barcelona 08193 Spain; ^5^ Emergent Molecular Function Research Group RIKEN Center for Emergent Matter Science (CEMS) Wako Saitama 351‐0198 Japan; ^6^ School of Chemistry and Biochemistry & Center for Organic Photonics and Electronics (COPE) Georgia Institute of Technology Atlanta GA 30332‐0400 USA; ^7^ Department of Chemistry and Biochemistry The University of Arizona Tucson AZ 85721‐0088 USA; ^8^ Dipartimento di Fisica Università di Cagliari, Cittadella Universitaria Monserrato (Ca) 09042 Italy

**Keywords:** molecular dynamics, organic semiconductors, thermal conductivity, thermoelectrics

## Abstract

While the charge transport properties of organic semiconductors have been extensively studied over the recent years, the field of organics‐based thermoelectrics is still limited by a lack of experimental data on thermal transport and of understanding of the associated structure–property relationships. To fill this gap, a comprehensive experimental and theoretical investigation of the lattice thermal conductivity in polycrystalline thin films of dinaphtho[2,3‐b:2′,3′‐f]thieno[3,2‐b]thiophene (C*n*‐DNTT‐C*n* with *n* = 0, 8) semiconductors is reported. Strikingly, thermal conductivity appears to be much more isotropic than charge transport, which is confined to the 2D molecular layers. A direct comparison between experimental measurements (3ω–Völklein method) and theoretical estimations (approach‐to‐equilibrium molecular dynamics (AEMD) method) indicates that the in‐plane thermal conductivity is strongly reduced in the presence of the long terminal alkyl chains. This evolution can be rationalized by the strong localization of the intermolecular vibrational modes in C8‐DNTT‐C8 in comparison to unsubstituted DNTT cores, as evidenced by a vibrational mode analysis. Combined with the enhanced charge transport properties of alkylated DNTT systems, this opens the possibility to decouple electron and phonon transport in these materials, which provides great potential for enhancing the thermoelectric figure of merit *ZT*.

## Graphical abstract

A significant drop in in‐plane thermal conductivity upon end alkylation of the aromatic rings of dinaphtho[2,3‐b:2′,3′‐f]thieno[3,2‐b] thiophene (DNTT), which is understood from a detailed vibrational‐mode analysis, is identified via a comprehensive experimental and theoretical investigation. Combined with their enhanced charge transport, the results suggest the possibility of decoupling electron and phonon transport in alkylated DNTT systems, which opens pathways for improved thermoelectric applications.

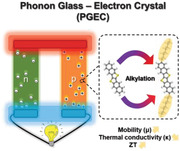

## 1 Introduction

Thermoelectric materials offer a simple solution for direct heat‐to‐electricity conversion from a variety of heat sources via the Seebeck effect. Worldwide, about 2/3 of primary energy is currently wasted as heat.^[^ ^1^ ^]^ Therefore, there exist great opportunities for enhancing the energy efficiency of many power generation and industrial processes. Current thermoelectric materials operate far from theoretical efficiency limits. There are intense ongoing research efforts to improve efficiency and enable wider applications in waste heat harvesting.^[^ ^2^, ^3^, ^4^ ^]^ One class of materials being explored for this purpose is that of organic semiconductors (OSCs).

The efficiency of a thermoelectric material is determined by the dimensionless figure of merit *zT = S* ^2^ *σT*/(κ_e_+ κ_ph_), where *S* [V K^–1^] denotes the Seebeck coefficient; σ [S m^–1^], the electrical conductivity; κ_e_ and κ_ph_ [W m^–1^ K^–1^], the electronic and phononic components of the thermal conductivity, respectively; and *T* [K], the absolute value of the average temperature between the cold and hot sides. For a good thermoelectric material, it is also desirable to decouple electron and phonon transport; the notion of an "electron crystal–phonon glass" originally proposed by Slack,^[^ ^5^ ^]^ in which electron mean‐free paths are long while phonon mean free paths are short, remains an important concept, whose implementation has been attempted in different classes of inorganic materials with skutterudites^[^ ^6^ ^]^ and clathrates^[^ ^7^ ^]^ being among the best‐known examples.

Herringbone‐stacked alkylated thienoacene‐based molecular materials have recently emerged as some of the best performing OSCs, a result of p‐type charge carrier mobilities that can reach over 10 cm^2^ V^–1^ s^–1^.^[^ ^8^, ^9^, ^10^ ^]^ Among this family, dinaphtho[2,3‐*b*:2′,3′‐*f*]thieno[3,2‐b]thiophene (DNTT) derivatives have been investigated in detail both experimentally and theoretically; the results point to a favorable 2D character of charge transport (i.e., within the layers).^[^ ^11^, ^12^, ^13^, ^14^, ^15^, ^16^, ^17^, ^18^ ^]^ Importantly, the origin of their high charge mobilities has recently been attributed to their reduced dynamic disorder, coupled to an isotropic electronic coupling pattern within the plane of charge transport. Indeed, alkylation has been demonstrated to: i) shift in‐plane phonon modes to higher frequencies, hence suppressing their impact on disorder; and ii) reduce the amplitude of the out‐of‐plane long‐axis sliding motions (the so‐called killer‐mode).^[^ ^19^, ^20^, ^21^ ^]^ Combined to good environmental stability and ease of processing through vapor and solution deposition techniques,^[^ ^22^ ^]^ these materials thus constitute ideal candidates for thermoelectric applications. However, a detailed investigation of their ability to transport heat has not been performed yet.

To date, only few studies have been dedicated to the thermal transport properties of OSCs.^[^ ^23^ ^]^ The thermal conductivity in organic materials is usually assumed to be low due to lattice disorder. More specifically, the limited thermal conductivity in such systems is attributed to localization of lattice vibrations described via the Einstein model of isolated atomic oscillations^[^ ^24^ ^]^ with heat being carried out through the lattice via random walk rather than through wavelike motions of collective oscillations.^[^ ^25^ ^]^ The exact mechanism of phonon localization, however, is not well understood. The Einstein‐like behavior was observed in small single crystals of alpha‐monoclinic selenium^[^ ^26^ ^]^ and, in spite of their microcrystallinity, in C_60_/C_70_.^[^ ^27^ ^]^ More recently, very low values of thermal conductivity were also found in layered WSe_2_ crystals – which was speculated to be caused by phonon localization induced by random stacking of 2D crystalline thin sheets^[^ ^28^ ^]^—and in the fullerene derivatives PCBM and PCBNB^[^ ^29^, ^30^ ^]^—which was explained, similarly to C_60_/C_70_, by localization of vibrational modes of the rigid buckyball molecules. The in‐plane and out‐of‐plane thermal conductivities of various systems (pentacene, Alq_3_, C_60_, rubrene, TIPS‐pentacene, CuPc)^[^ ^31^, ^32^, ^33^, ^34^, ^35^, ^36^ ^]^ with sample ranging from polycrystals to single crystals, have been measured macroscopically using techniques such as ac‐calorimetry, the 3ω method or time domain thermoreflectance measurements and have been found to be in the range 0.1–0.8 W m^–1^ K^–1^. However, a detailed understanding of structure–property relationships remains elusive.

One reason for the lack of studies of the thermal transport properties is that thermal conductivity is a challenging transport coefficient to measure reliably. Thermal conductivity κ is defined according to Fourier's heat conduction equation: $\dot{Q}=\;\;-\kappa \frac{\Delta T}{\Delta l}$, where $\dot{Q}$ [W m^–2^] is the heat flux passing through the sample; Δ*l* [m], the sample length; and Δ*T* [K], the temperature difference across the sample. Parasitic heat transfer through radiative (infrared) heat exchange with the surroundings and losses due to thermal resistance at interfaces present in virtually every experimental system create high uncertainty in the measured heat flux values.^[^ ^37^, ^38^ ^]^ Another unexpectedly large uncertainty in thermal conductivity measurements comes from measurements of sample dimensions, as has been demonstrated by an international round‐robin testing of bulk thermoelectric materials.^[^ ^39^ ^]^ As a result, even for measurement setups designed in accordance with well‐established measurement standards, the combined measurement uncertainty can reach up to 20% for bulk materials. Reduced sample dimensions in the case of thin films worsen the situation and the experiments require extra care at every step to keep the overall uncertainty within acceptable limits.

## 2 Results and Discussion

**Figure 1:**
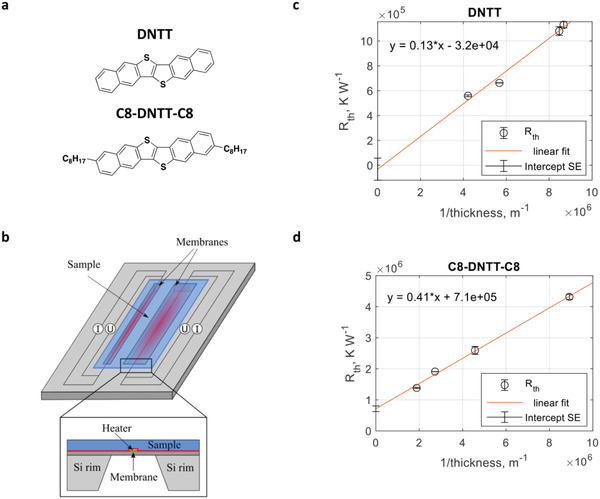
Molecular structures of the investigated materials and experimental details of the thermal conductivity measurements implementing the 3ω–Völklein method. a) Molecular structures of the investigated materials; i.e., DNTT (top) and C8‐DNTT‐C8 (bottom). b) Schematic description of the measurement setup. In a cross‐sectional view (inset) the interface across which thermal resistance can contribute to measurement uncertainty is marked in red. c) Thermal resistance of DNTT and d) C8‐DNTT‐C8 as inverse function of their thickness. The intercepts with the *y*‐axis are used to estimate the contribution from the thermal contact resistance at the interface between the film and the substrate; the corresponding error bars represent the standard errors of the linear regression.

In this work, we report a comprehensive experimental and theoretical investigation of the lattice thermal conductivity in polycrystalline thin films of dinaphtho[2,3‐b:2′,3′‐f]thieno[3,2‐*b*]thiophene (C*n*‐DNTT‐C*n* with *n* = 0, 8) semiconductors (the molecular structures are given in **Figure** **1**a). Considering the anisotropy of the material properties and the fact that DNTT molecules tend to have a preferential orientation with their long molecular axis nearly perpendicular to the substrate plane, it is important to distinguish between the in‐ and out‐of‐plane transport directions. Wang et al.^[^ ^23^ ^]^ measured the thermal conductivity of DNTT thin films in the out‐of‐plane direction with the traditional 3ω‐method.^[^ ^40^ ^]^ However, in view of the potential thermoelectric applications, the thermal transport in the in‐plane direction of thin films of small‐molecule semiconductors is more relevant since it aligns with the direction of fast electrical transport in these materials. Here, we measured the in‐plane thermal conductivity of undoped DNTT and its alkylated derivative C8‐DNTT‐C8 following our previously established protocol that reduces measurement uncertainty.^[^ ^41^ ^]^ The experimental setup is schematically presented in Figure 1b.

Thermal conductivity measurements were performed in the in‐plane direction according to the 3ω–Völklein method^[^ ^42^, ^43^ ^]^ implemented in a commercial Linseis Thin Film Analyzer (TFA).^[^ ^44^ ^]^ More information about the method and its advantages and can be found in the Supporting Information.

The samples were prepared through thermal evaporation of a DNTT layer on top of the membrane of the measurement chips using a masking shutter that allows the deposition of four different thicknesses within the same conditions. Adequate masking insured specific deposition over the active area. Grazing‐incidence wide‐angle X‐ray scattering (GIWAXS) and atomic force microscopy (AFM) investigations allowed us to analyze the film microstructures and surface morphologies as well as to determine their respective thicknesses.

The maximum thickness of the DNTT films was ≈240 nm and all films showed a polycrystalline structure with a preferred orientation where the long molecular axis is nearly perpendicular to the substrate plane. In the case of C8‐DNTT‐C8 films, the growth rate was enhanced and the maximum thickness was ≈ 530 nm (for similar deposition conditions, see Experimental Section). The thinnest C8‐DNTT‐C8 film showed a preferred orientation while thicker C8‐DNTT‐C8 films showed powder‐like distribution of crystallites (causing rings in the GIWAXS data) and only a slightly preferred orientation of the crystallites (peaks on rings), pointing to an edge‐on orientation as in the case of DNTT (Figure S3, Supporting Information). The AFM topography for the C8‐DNTT‐C8 films also revealed larger features and a higher surface roughness (Figure S4, Supporting Information). In spite of these differences, the average crystallite size was found to be the same for all samples in the study, in the range of ≈7–10 nm (Table S1, Supporting Information).

As pointed out previously, parasitic heat losses at interfaces with thermal contact resistances are among the major sources of uncertainties in thermal conductivity measurements. One of the interfaces where thermal contact effects are likely to arise in the present setup is that between the sample film and the membrane (marked in red in Figure 1b (inset)). The contribution from this interface can be estimated by plotting the apparent thermal resistance, $R_{\text{th}}\;\;=\;\;\frac{\Delta T}{Q}$ [K W^–1^], with respect to the sample thickness and approximating the intercept of the curve with the *y*‐axis.^[^ ^41^ ^]^ For all samples under investigation, the total thermal resistance (sample + membrane) was clearly separable from that of the empty membrane, indicating a clear contribution of the sample to the thermal response (Figure S5, Supporting Information).

The evolutions of *R*_th_ versus sample thickness are presented in Figure 1c,d. Note that since the direction of heat transport is in the plane of the sample film, the sample thickness defines the cross‐sectional area of heat transfer, not the sample length. The effective thermal resistance is thus plotted versus inverse sample thickness. Linear extrapolation of the intercepts with the *y*‐axis including the uncertainty of the linear regression resulted in small, yet not negligible values. However, the clear linear trends for both DNTT and C8‐DNTT‐C8, consistent with the scaling sample cross‐section, indicated that the apparent thermal resistance is dominated by the material property and that the offset is likely caused by the uncertainties associated with the repeatability of thermal conductivity measurements and film thickness determination.

**Figure 2:**
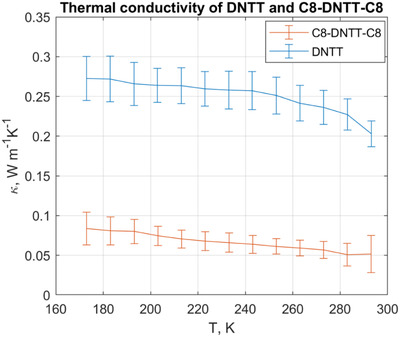
Measured in‐plane thermal conductivities of thin films of DNTT and C8‐DNTT‐C8 as a function of temperature. The error bars represent the combined uncertainty associated with standard deviation in the repeatability of measurements due to variability in thermal contacts and other experimental factors and standard deviation associated with the thickness determination (including film non‐uniformity and surface roughness).

The temperature evolutions of the thermal conductivities of DNTT and its alkylated derivative, C8‐DNTT‐C8, are presented in **Figure** **2**. Due to high thermal resistance of the films under investigation, we were able to obtain reliable data only from the effective sample area on top of the smaller membrane; as a result, it was not possible to correct for the radiative losses in these experiments. Thus, the reported data present an apparent thermal conductivity that includes thermal conduction through the material and conduction through infrared radiation; hence, the values are overestimated.

The thermal conductivity decreases with temperature, which is consistent with the trend found in crystalline materials.^[^ ^45^ ^]^ The decrease may appear statistically insignificant in case of C8‐DNTT‐C8; however, the trend is more evident when the error bars do not include the standard deviation associated with the thickness determination (Figure S6, Supporting Information). Since the film thickness is not expected to change significantly as a function of temperature (substantial thermal expansion and material degradation typically occur at higher temperatures (above 100 °C^[^ ^46^, ^47^ ^]^)), this standard deviation would cause an offset in the mean thermal conductivity value without changing the overall trend. We note that in the case of C8‐DNTT‐C8 samples, this trend was not found in the thicker films presenting only a slightly preferred orientation of crystallites, compared to the thinnest film for which the preferential alignment was clearly demonstrated (Figure S6, Supporting Information). The most striking result, however, is the extremely low thermal conductivity of the alkylated derivative C8‐DNTT‐C8 compared to its nonalkylated counterpart. Since the C8‐DNTT‐C8 film with the preferred orientation of crystallites had similarly low thermal conductivity, the thermal conductivity reduction cannot be correlated solely with the decrease in the preferential alignment of crystallites. We also note that despite the complications due to orientational changes with increasing thickness, the thermal resistance as a function of inverse thickness exhibit a clear linear behavior across the entire thickness range (Figure 1c,d) for both molecules. This also suggests that orientational effects are not the dominant factor when comparing the thermal conductivity extracted for the two molecules. The room temperature value of ≈0.05 W m^–1^ K^–1^ is comparable to the ultralow thermal conductivities observed in a few systems, such as C_60_/C_70_,^[^ ^27^ ^]^ layered WSe_2_^[^ ^28^ ^]^ crystals, and the fullerene derivatives PCBM and PCBNB.^[^ ^29^, ^30^ ^]^

Our experimental results thus suggest that the in‐plane thermal conductivity is significantly reduced by adding alkyl chains on the terminal aromatic rings. Indeed, such observations are convincingly strengthened by the following theoretical estimations. Herein, we focused exclusively on the lattice thermal conductivity since the electronic contribution to the thermal transport is expected to be weak in neutral or slightly doped OSCs,^[^ ^48^ ^]^ see the discussion in the Supporting Information; all our experimental data were collected on undoped samples. Owing to the air stability of DNTT‐based materials,^[^ ^22^ ^]^ we do not expect significant unintentional doping by environmental contaminants (O_2_ and H_2_O). This is supported by our experience with FET devices made with the two molecules which generally exhibit very low OFF currents.^[^ ^21^ ^]^

We estimated the lattice thermal conductivity in single crystals made of DNTT cores and their alkylated derivatives C8‐DNTT‐C8 via the "approach‐to‐equilibrium molecular dynamics" (AEMD) method.^[^ ^49^ ^]^ In essence, AEMD computes the lattice thermal conductivity of a material based on the rapid decay time of a thermal gradient initially created along a specific crystal orientation. More precisely, this property is deduced from the exponential fit of the time‐decreasing temperature difference between the hot and cold region of the simulation box with an appropriate solution of the 1D heat equation:^[^ ^49^ ^]^ $\frac{\partial T}{\partial t}\;\;=\;\;D\frac{\partial^{2}T}{\partial x^{2}}$. Therefore, the lattice thermal conductivity, κ [W m^–1^ K^–1^], can be straightforwardly derived from the thermal diffusivity, *D*  =  κ/*ρC*_p_ [m^2^ s^–1^], provided that the mass density, ρ [kg m^–3^], and heat capacity, *C*_p_ [J kg^–1^ K^–1^], of the system are well defined. Here, we relied on the Dulong–Petit model,^[^ ^50^ ^]^ which considers the specific heat capacity *C*_p_ to be strictly equal to *3R*. Further description of the method, its advantages as well as an example of a typical AEMD simulation can be found in the Supporting Information.

**Figure 3:**
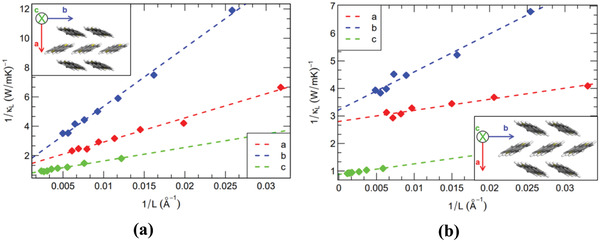
a,b) Inverse of the lattice thermal conductivity as a function of the inverse of the box length along directions *a*, *b* and *c* for DNTT (a) and C8‐DNTT‐C8 (b). Each inset represents the layered herringbone packing of DNTT and C8‐DNTT‐C8 in the *ab* plane. For the sake of clarity, the alkyl chains have been omitted in the inset of part (b).

The inverse of the lattice thermal conductivity versus the inverse of the length of the supercell along the three major crystal axes of DNTT and C8‐DNTT‐C8 is shown in **Figure** **3**. We note that these two organic compounds exhibit a monoclinic structure (with *a* = 6.187 Å, *b* = 7.662 Å, *c* = 16.210 Å, α = 92.490° for DNTT and *a* = 5.987 Å, *b* = 7.861 Å, *c* = 34.066 Å, β = 99.860° for C8‐DNTT‐C8; respectively^[^ ^21^ ^]^) and a herringbone packing motif, which is essentially maintained by CH–π interactions in the *ab* plane, with the *c*‐axis lying perpendicular with respect to the substrate plane. A linear regression through the data gives lattice thermal conductivity values of 0.79, 0.73, and 1.40 W m^–1^ K^–1^ along directions *a*, *b*, and *c* for DNTT and 0.35, 0.31, and 1.14 W m^–1^ K^–1^ along the same directions for C8‐DNTT‐C8. In the latter case, our results are consistent with those of the theoretical work of Shi et al.,^[^ ^51^ ^]^ who have reported very similar thermal conductivity values in the *ab* plane by using the NEMD method for the study of dioctyl[1]benzothieno[3,2‐b][1]‐benzothiophene (C8‐BTBT‐C8). The thermal conductivity ratios *a*/*b*, *c*/*a*, and *c*/*b* are 1.08, 1.77, and 1.92 for DNTT and 1.13, 3.26, and 3.68 for its alkylated derivative. Of notable interest is that the phonon mean free paths (MFPs), *l*_bulk_ [Å], can be extracted from the analytical equations associated with the extrapolation curves. Without dwelling on theoretical concepts that have already been discussed in detail elsewhere,^[^ ^52^, ^53^, ^54^ ^]^ we simply mention that such a deduction is made possible by combining the kinetic formulation of the thermal conductivity (namely, $\kappa \;\;=\;\;\frac{1}{3}\;\rho C_{p}\tau_{\text{bulk}}^{2}l_{\text{bulk}}$; where τ_bulk_ [fs] is the bulk phonon relaxation time) with the decomposition of τ_bulk_ into separate terms due to the independence of the various scattering events arising in the system, as predicted by the Matthiessen's rule. Hence, the average phonon MFPs along directions *a*, *b*, and *c* are 129.0, 293.0, and 129.0 Å for DNTT and 15.0, 43.6, and 44.0 Å for C8‐DNTT‐C8, respectively. Unexpectedly, in contrast to the generally 2D character of charge transport in molecular semiconductors with very weak electronic coupling between molecules lying in adjacent layers,^[^ ^11^, ^12^, ^13^, ^55^, ^56^ ^]^ the computed thermal conductivity appears to be much more isotropic, with the most efficient direction for heat transport actually corresponding to the interlayer axis. Since the 3ω–Völklein method used experimentally on the polycrystalline samples provides an isotropically averaged value through the *ab* plane, we have defined in turn an average thermal conductivity as κ_in_ = (κ_a_ + κ_b_)/2 for ease of comparison with the experimental data. On that basis, the calculations indicate that the thermal conductivity is reduced within the layers upon alkylation of the DNTT core, which is fully consistent with the experimental results. In addition, this structure–property relationship is consistent with a recent joint experimental and theoretical investigation of thermal transport properties of nonalkylated and alkylated BTBT derivatives probed by thermal scanning microscopy along the out‐of‐plane (interlayer) direction.^[^ ^57^ ^]^ From a quantitative perspective, the effects of alkylation of DNTT lead to a decrease in the calculated in‐plane thermal conductivity by a factor of ≈2.3, with this drop being experimentally even more pronounced (a factor of ≈ 4). As mentioned earlier, our experimental data on the thermal conductivity of C8‐DNTT‐C8 are very similar to those observed in the state‐of‐the‐art fullerene derivatives PCBM and PCBNB; such a low thermal conductivity could be of great interest for the development of efficient thermoelectric applications. We also draw attention to the fact that the calculated in‐plane thermal conductivities κ_in_ are 3.8 and 6.6 times higher for DNTT and C8‐DNTT‐C8 when compared to the corresponding experimental measurements. The origin of these overestimations could be threefold: i) the predominance of the harmonic approximation in the expression of the potential energy (i.e., for bonds and angles) while anharmonic terms would yet better account for phonon–phonon interactions; ii) the occurrence of scattering processes at grain boundaries due to the polycrystalline nature of the organic thin films, as confirmed by the GIWAXS experiments; and/or iii) the presence of impurities that can strongly affect thermal transport by acting as phonon scattering centers. Another source of overestimation could arise from the absence of quantum corrections in our calculations; nevertheless, the Dulong–Petit model^[^ ^50^ ^]^ can be considered as valid since the MD simulations are conducted at room temperature while the Debye temperature θ_D_ of many OSCs rarely exceeds 100 K.^[^ ^58^, ^59^ ^]^ It is worth noting that Wang et al.^[^ ^23^ ^]^ reported an out‐of‐plane thermal conductivity κ_out_ = 0.45 ± 0.06 W m^–1^ K^–1^ for a DNTT thin film with a thickness of 50 nm, as measured at room temperature by means of the differential 3ω method. By combining this value with the current experimental data, we obtain an anisotropy factor $\kappa_{\text{out}}^{\exp}/\kappa_{\text{in}}^{\exp}$ ≈ 2.5, which is very similar to the corresponding theoretical ratio $\kappa_{\text{out}}^{\text{theo}}/\kappa_{\text{in}}^{\text{theo}}$ ≈ 1.9, even though it must be borne in mind that the values of both in‐plane and out‐of‐plane thermal conductivities are overestimated by our theoretical calculations.

**Figure 4:**
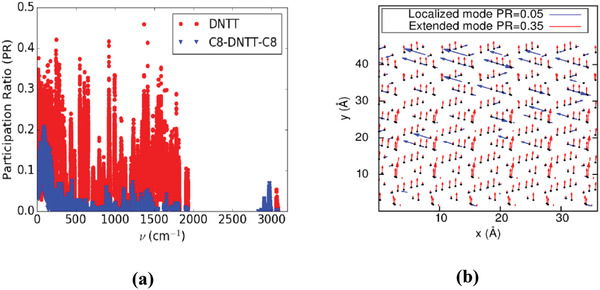
a) Estimated participation ratio (PR) as a function of frequency for DNTT (red) and C8‐DNTT‐C8 (blue). b) Atomic displacement fields for two eigenmodes in a section of DNTT with thickness of 5 Å. Displacements (properly scaled) are shown as blue [red] vectors for localized [extended] modes with PR = 0.05 [0.35] respectively.

The origin of such a strong suppression of thermal transport in the alkylated derivatives can be explained via the estimation of the participation ratio (PR) of the lattice vibrational modes.^[^ ^60^ ^]^ As described in the Experimental Section, this parameter is derived from the diagonalization of the dynamical matrix and offers a quantitative estimate of the spatial extension of each vibrational mode. More specifically, it allows to distinguish between extended modes (wavelike motions or collective oscillations), characterized by a large PR value (>0.4), and localized modes (Einstein model of isolated atomic oscillations), which have a PR close to zero. The calculated participation ratios in DNTT and C8‐DNTT‐C8 are shown in **Figure** **4**a as a function of frequency. As can be seen, the DNTT participation ratios are found to be systematically larger than those in C8‐DNTT‐C8 over the whole frequency window. This underscores the critical role played by long alkyl chains in strongly localizing the lattice modes in C8‐DNTT‐C8 with respect to DNTT. The localized character of the modes is expected to hinder their capability of transmitting heat, hence leading to a pronounced decrease in the total in‐plane thermal conductivity. A more intuitive understanding of the difference between extended and localized modes is gained by looking at their atomic displacements. To this aim, we show in Figure 4b the displacement field of a high‐frequency localized mode with PR = 0.05 and a low‐frequency extended mode with PR = 0.35 in a section of DNTT with thickness of 5 Å. While in the extended mode almost all atoms participate in the motion, in the localized mode only a small subset of the atoms is significantly vibrating around the equilibrium positions.

## 3 Conclusions

We have conducted a detailed experimental and theoretical study of the thermal transport properties in the crystalline structure of nonalkylated and alkylated DNTT derivatives. Thermal conductivity measurements were performed with the 3ω–Völklein technique following a protocol with reduced measurement uncertainty. On the theoretical side, we have exploited the robustness and accuracy of the AEMD method to estimate the phononic component of the heat diffusion along their crystal lattices. Our results emphasize the critical impact of establishing robust structure–property relationships in order to design the phonon characteristics in organic semiconductors and hence their heat transport properties. A direct comparison between experimental measurements and theoretical estimations highlights a noticeable drop of the in‐plane thermal conductivity upon addition of long alkyl side chains to the aromatic rings, which were found to disrupt the collective motion of the atoms, leading to reduction of the spatial extension of the vibrational modes, i.e., mode localization. This drop ultimately leads to remarkably low experimental thermal conductivities on the order of 0.05 W m^–1^ K^–1^, comparable to the lowest values experimentally observed to date. Since, among the two systems investigated here, C8‐DNTT‐C8 is known from field‐effect transistor (FET) studies to exhibit higher carrier mobilities, our results suggest the possibility of decoupling electron and phonon transport in alkylated DNTT systems and realizing the so‐called “phonon glass–electron crystal" concept, which is of high interest for enhancing the thermoelectric figure of merit *ZT*. The combination of molecular design and accurate investigations of charge and thermal transport properties will open new pathways for improved thermoelectric applications.

## 4 Experimental Section

### Materials

DNTT was purchased from Sigma‐Aldrich and used as received. C8‐DNTT‐C8 was synthesized according to previously described procedures and supplied by Nippon Kayaku.^[^ ^15^, ^61^ ^]^

The organic thin films were deposited by thermal evaporation (Angstrom Engineering, Inc.) at a rate of 0.5 Å s^–1^ under the pressure of 10^–7^ Torr and substrate rotation of 25 rpm, using a masking shutter.

### Calculation of the Lattice Thermal Conductivity

The MD simulations were performed with the Large‐scale Atomic/Molecular Massively Parallel Simulator (LAMMPS) package.^[^ ^62^ ^]^ The systems are described with a properly reparametrized version of the OPLS‐AA force field whose bonded and nonbonded parameters are implemented in the LAMMPS code via the LigParGen server.^[^ ^63^ ^]^ The localized bond‐charge corrected CM1A (1.14*CM1A‐LBCC) charge model is employed.^[^ ^64^ ^]^ We have verified that this methodology generates thermal molecular motions around the crystal equilibrium geometry, without spurious drifts typically observed with raw force fields. Moreover, simulations with a properly tuned force field are able to depict reliably the low frequency modes of interest for thermal conductivity when compared to corresponding quantum‐chemical calculations.^[^ ^65^ ^]^ The DNTT and C8‐DNTT‐C8 unit cells are elongated along each direction of interest for analyzing the heat transport and are then replicated using periodic boundary conditions. These supercells are first optimized at constant lattice parameters before relaxing the box dimensions during a second optimization step. Next, the systems are successively equilibrated in the NVT and then in the NPT ensemble, using a velocity‐Verlet algorithm to solve the equation of motions and the Nosé–Hoover thermostat and barostat to monitor the temperature and the pressure. Each simulation lasts for 1 ns under standard temperature and pressure conditions with a timestep of 1.0 fs, using 10 Å and 12 Å as van der Waals and electrostatic cutoff distances, respectively. In the AEMD approach, a controlled step‐like temperature profile is applied by keeping fixed atomic positions in one half of the simulation box while consecutively thermostating at <*T*_1_> = 362.5 K and <*T*_2_> = 237.5 K the other half of the system during two short NVT simulations. An NVE simulation up to 3 ns is then needed to dissipate the initial *ΔT* = <*T*_1_> – <*T*_2_> = 125 K thermal gradient. Finally, a size‐dependent lattice thermal conductivity is obtained by reinjecting the following fitting constant $\sum_{5}^{n\;=\;1}C_{n}e^{-\alpha_{n}^{2}Dt}$ into the 1D heat equation.^[^ ^49^ ^]^ Note that *C_n_* and α_*n*_ are coefficients depending on the size of the supercell, the integer *n* and the initially imposed temperature gradient.

### Estimation of the Participation Ratio

We studied the vibrational properties of both DNTT and C8‐DNTT‐C8 by setting up and diagonalizing the dynamical matrix of one snapshot of the samples, given by:

(1) $$D_{i\alpha ,j\beta }\;\;=\;\;\frac{-1}{\sqrt{m_{i}m_{j}}}\;\frac{\partial F_{i\alpha }}{\partial r_{j\beta }}$$

In this equation, the Greek letters indicate the (*x*,*y*,*z*) Cartesian components while the Latin indices are used for labeling atoms. Here *m_i_* is the mass of the *i*th atom. *F_iα_* is the force on the *i*th atom along direction α and *r_jβ_* an infinitesimal displacement of atom *j* along direction β.

The calculation of the first derivative in the above equation has been performed by finite difference with an atomic displacement of 5 × 10^−4^. The dynamical matrix has then been diagonalized by means of the SLEPc library^[^ ^66^ ^]^ by obtaining the eigenvectors *e_s_* and eigenvalues ω^2^ *_s_* where *s* = 1, . . ., 3*N* counts eigenmodes.

The participation ratio (PR) is finally estimated as:^[^ ^67^ ^]^

(2) $$\text{PR}\;\;=\;\;\frac{1}{N}\;\frac{{\left(\sum {\underset{i\;=\;1}{\overset{N}{e_{i,s}}}}^{2}\right)}^{2}}{\sum {\underset{i\;=\;1}{\overset{N}{e_{i,s}}}}^{4}}$$

providing a normalized estimation of the subgroup of atoms involved in the *s*‐th vibrational mode. The spatial extension of such a subgroup is linked to the localized or extended nature of that mode: for extended modes PR ≈ 1, while localized modes have a smaller ratio, down to the limit PR = 1/*N* for a mode completely localized on a single atom. It is worth noticing that, according to its very definition, a PR exactly equal to unity is obtained solely for vibrational modes in ideal crystalline systems, in which the atomic displacements are perfectly periodic within the sample. In turn, its value rapidly decreases if tiny inhomogeneities are present in the atomistic coordinates or as a consequence of a possible numerical uncertainty in the atomic displacements. In both cases, the extended character of the vibrational modes is preserved. Calculated values of PR for extended modes in noncrystalline systems lie in the range [0.4–0.6].

### Atomic Force Microscopy (AFM)

The measurements were performed with an MFP‐3D AFM System (Asylum/Oxford Instruments) in AC (noncontact) mode. The samples thickness was obtained by calculating an average step height in the topography scans taken at the sample edges in the proximity of the four corners.

### Grazing‐Incidence Wide‐Angle X‐ray Scattering (GIWAXS)

GIWAXS measurements were performed using a Xeuss 2.0 SAXS/WAXS system (Xenocs) with a Dectris Pilatus3R 300 K detector using a wavelength of 1.5406 Å and an angle of incidence of 0.2°. For the measurements, the sample was placed in a vacuum chamber to reduce air scattering.

### Thermal Conductivity Evaluation

Calculations were performed according to Linseis et al.^[^ ^44^ ^]^ from the raw data obtained on Linseis TFA.

## Conflict of Interest

The authors declare no conflict of interest.

## Supporting information

Supporting Information

## Data Availability

The data that support the findings of this study are openly available in Apollo—The University of Cambridge Repository at https://doi.org/10.17863/CAM.68874.
