# Supplementary material for: Strong Suppression of Thermal Conductivity in the Presence of Long Terminal Alkyl Chains in Low‐Disorder Molecular Semiconductors
Source: Adv Mater. 2021 Aug 3;33(37):2008708. doi: 10.1002/adma.202008708 (PMC11468527; doi:10.1002/adma.202008708)
Supplement: Supplementary file 1 — Supporting Information [file ADMA-33-2008708-s001.pdf]

© 2021 Wiley-VCH GmbH

ADVANCED MATERIALS

Supporting Information

for *Adv. Mater.,* DOI: 10.1002/adma.202008708

Strong Suppression of Thermal Conductivity in the Presence of Long Terminal Alkyl Chains in Low-Disorder Molecular Semiconductors

Ekaterina Selezneva, Alexandre Vercouter, Guillaume Schweicher, Vincent Lemaur, Katharina Broch, Aleandro Antidormi, Kazuo Takimiya, Veaceslav Coropceanu, Jean-Luc Brédas, Claudio Melis, Jérôme Cornil,* and Henning Sirringhaus**

Strong Suppression of Thermal Conductivity in the Presence of Long Terminal Alkyl Chains in Low-Disorder Molecular Semiconductors

Ekaterina Selezneva[†1], Alexandre Vercouter[†2], Guillaume Schweicher[†1], Vincent Lemaur[2], Katharina Broch[3], Aleandro Antidormi[4], Kazuo Takimiya[5], Veaceslav Coropceanu[6], Jean-Luc Brédas[6,7], Claudio Melis*[8], Jérôme Cornil*[2], Henning Sirringhaus*[1]

[1] Optoelectronics Group, Cavendish Laboratory, University of Cambridge, JJ Thomson Avenue, Cambridge CB3 0HE, United-Kingdom

[2] Laboratory for Chemistry of Novel Materials, University of Mons, 7000 Mons, Belgium

[3] Institut für Angewandte Physik, Universität Tübingen, Aufder Morgenstelle 10, 72076 Tübingen, Germany

[4] Catalan Institute of Nanoscience and Nanotechnology (ICN2), CSIC and BIST, Campus UAB, Bellaterra, 08193, Barcelona, Spain

[5] Emergent Molecular Function Research Group, RIKEN Center for Emergent Matter Science (CEMS), Wako, Saitama, Japan

[6] School of Chemistry and Biochemistry & Center for Organic Photonics and Electronics (COPE), Georgia Institute of Technology, Atlanta, Georgia, 30332-0400, USA

[7] Department of Chemistry and Biochemistry, The University of Arizona, Tucson, Arizona 85721-0088, USA

[8]Dipartimento di Fisica, Universit`a di Cagliari Cittadella Universitaria, 09042 Monserrato (Ca), Italy

[†]These authors contributed equally to this work.
*e-mail: claudio.melis@dsf.unica.it; jerome.cornil@umons.ac.be; hs220@cam.ac.uk

S1. Thermal conductivity measurements: 3ω–Völklein method

In essence, the Völklein method allows correction for radiative losses by calculating and subtracting their contribution to the total heat conduction. This is achieved by performing measurements on two different geometries with different active areas integrated on the same substrate. This results in a system of two heat conduction equations with two unknowns: thermal conductivity and emissivity, which is then solved numerically.

In the measurement setup, the film is deposited on top of a specially designed silicon-based chip containing two free standing Si_3N_4 membranes of different area. Effective heat sinking over the silicon rim isolates two measurement areas of the sample film on top of the membranes. Two microfabricated wires aligned with the longitudinal axes of the membranes serve as heaters and resistive thermometers. A high aspect ratio of the membranes (and hence of the effective sample areas) ensures that the heat flux is predominantly one-dimensional – in the plane of the membranes and perpendicular to the heater wires[1]. Thus, the measurement probes the in-plane thermal conductivity of the sample, which is derived by a differential method. It involves subtracting the contribution from the empty membrane from the total thermal response of the membrane and sample.

Similarly to the traditional 3ω–method[2], the heating is produced by applying AC current of frequency ω to the heater wire connected in a 4-point-probe configuration. This results in Joule heating and corresponding oscillations in the wire resistance at frequency 2ω. Combined with the heating current, this produces a voltage component at frequency 3ω proportional to the temperature rise in the system. The temperature rise in a material is proportional to the thermal penetration depth defined as $d = \sqrt{\frac{2D}{\omega}}$, where D [m^2 s^{-1}] is the thermal diffusivity of the material; and ω is the angular frequency of the heat source. In such a case, the thermal conductivity can thus only be determined indirectly through the thermal diffusivity, according

to the following equation: $\kappa = D\rho C_p$, which requires additional measurements of the material density, ρ [kg m^{-3}]; and heat capacity, C_p [J kg^{-1} K^{-1}]. The uncertainty of the thermal conductivity value will thus include the uncertainties of the measurements of these physical quantities. The uncertainty of heat capacity measurements alone could be as high as 15%, as was observed in the international round-robin study with all the laboratories following the same specific guide lines[3]. However, when the thermal penetration depth exceeds the sample dimensions, the temperature response in the material becomes frequency independent. These so-called 'quasi steady-state' conditions are implemented in the 3ω–Völklein method by applying an AC current at very low frequencies of ~ 0.4 – 0.5 Hz; then, the thermal penetration depth exceeds the membrane width, thus the effective sample dimension. This allows for the determination of the thermal conductivity directly without additional measurement of the material density and heat capacity.

S2. OPLS-AA force-field parameters (in LAMMPS format) for the C8-DNTT-C8 molecule:

(a)

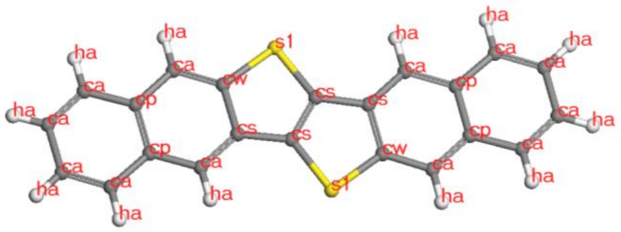

(b)

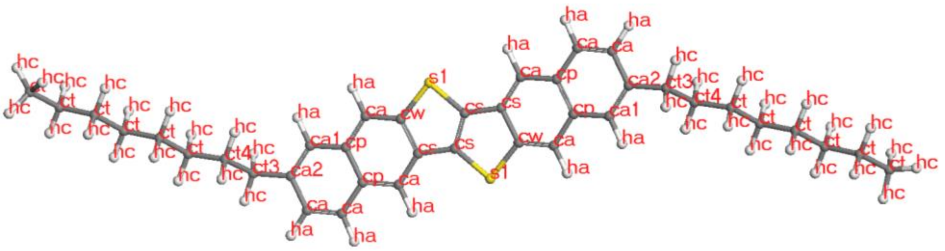

Figure S1. OPLS-AA force-field types defined for the (a) DNTT and (b) C8-DNTT-C8 molecules.

Masses

```
 1  12.010700 # ca1
 2  12.010700 # ca2
 3  12.010700 # ca
 4  12.010700 # cp
 5  12.010700 # cs
 6  12.010700 # ct3
 7  12.010700 # ct4
 8  12.010700 # ct
 9  12.010700 # cw
10   1.007940 # ha
11   1.007940 # hc
12  32.065000 # s1
```

Pair Coefficients

```
1 0.070000    3.550000    0.035000    3.550000 # ca1
2 0.070000    3.550000    0.035000    3.550000 # ca2
3 0.070000    3.550000    0.035000    3.550000 # ca
4 0.080000    3.500000    0.040000    3.500000 # cp
5 0.070000    3.550000    0.035000    3.550000 # cs
```

6 0.066000 3.500000 0.033000 3.500000 # ct3
7 0.066000 3.500000 0.033000 3.500000 # ct4
8 0.066000 3.500000 0.033000 3.500000 # ct
9 0.080000 3.500000 0.040000 3.500000 # cw
10 0.030000 2.420000 0.015000 2.420000 # ha
11 0.030000 2.500000 0.015000 2.500000 # hc
12 0.355000 3.600000 0.177500 3.600000 # s1

Bond Coefficients

1 469.0 1.4000 # ca1-ca2
2 469.0 1.4040 # cp-ca1
3 367.0 1.0800 # ha-ca1
4 469.0 1.4000 # ca2-ca
5 317.0 1.5100 # ct3-ca2
6 469.0 1.4000 # ca-ca
7 469.0 1.4040 # ca-cp
8 469.0 1.4000 # ca-cs
9 469.0 1.4040 # cw-ca
10 367.0 1.0800 # ha-ca
11 520.0 1.3700 # cp-cp
12 469.0 1.4240 # cs-cs
13 469.0 1.4240 # cw-cs
14 250.0 1.7600 # cs-s1
15 268.0 1.5290 # ct4-ct3
16 340.0 1.0900 # hc-ct3
17 268.0 1.5290 # ct-ct4
18 340.0 1.0900 # hc-ct4
19 268.0 1.5290 # ct-ct
20 340.0 1.0900 # hc-ct
21 250.0 1.7600 # cw-s1

Angle Coefficients

1 63.0 120.00 # ca-ca2-ca1
2 70.0 120.00 # ca1-ca2-ct3
3 85.0 134.90 # ca1-cp-ca
4 85.0 117.30 # cp-cp-ca1
5 63.0 120.00 # ca2-ca1-cp
6 35.0 120.00 # ca2-ca1-ha
7 63.0 120.00 # ca-ca-ca2
8 35.0 120.00 # ca2-ca-ha
9 63.0 114.00 # ca2-ct3-ct4
10 35.0 109.50 # ca2-ct3-hc
11 70.0 120.00 # ca-ca2-ct3
12 63.0 120.00 # cp-ca-ca
13 35.0 120.00 # ca-ca-ha
14 85.0 134.90 # ca-cp-ca
15 85.0 117.30 # ca-cp-cp
16 63.0 120.00 # cs-cs-ca

17 63.0   120.00 # ca-cs-cw
18 85.0   134.90 # cs-cw-ca
19 85.0   119.40 # s1-cw-ca
20 35.0   120.00 # cp-ca1-ha
21 63.0   120.00 # cs-ca-cp
22 63.0   120.00 # cp-ca-cw
23 35.0   120.00 # cp-ca-ha
24 35.0   120.00 # cs-ca-ha
25 63.0   120.00 # cs-cs-cs
26 70.0   107.30 # cs-cs-cw
27 85.0   119.40 # s1-cs-cs
28 85.0   119.40 # s1-cw-cs
29 63.0   112.40 # cw-s1-cs
30 58.4   112.70 # ct3-ct4-ct
31 37.5   110.70 # ct3-ct4-hc
32 37.5   110.70 # ct4-ct3-hc
33 58.4   112.70 # ct4-ct-ct
34 37.5   110.70 # ct4-ct-hc
35 37.5   110.70 # ct-ct4-hc
36 58.4   112.70 # ct-ct-ct
37 37.5   110.70 # ct-ct-hc
38 35.0   120.00 # cw-ca-ha
39 33.0   107.80 # hc-ct3-hc
40 33.0   107.80 # hc-ct4-hc
41 33.0   107.80 # hc-ct-hc

Dihedral Coefficients

1 0.000   7.250   0.000   0.000 # ca1-ca2-ca-ca
2 0.000   7.250   0.000   0.000 # ha-ca-ca2-ca1
3 -0.20   -0.100   -0.350   0.030 # ct4-ct3-ca2-ca1
4 0.000   0.000   0.000   0.000 # hc-ct3-ca2-ca1
5 0.000   7.250   0.000   0.000 # ca1-cp-cp-ca
6 0.000   7.000   0.000   0.000 # ca-cp-ca1-ca2
7 0.000   7.000   0.000   0.000 # ca2-ca1-cp-cp
8 0.000   7.250   0.000   0.000 # ca2-ca-ca-cp
9 0.000   7.250   0.000   0.000 # ha-ca-ca-ca2
10 1.300   -0.050   0.200   0.000 # ct-ct4-ct3-ca2
11 0.000   0.000   0.462   0.000 # hc-ct4-ct3-ca2
12 0.000   0.000   0.000   0.000 # ct4-ct3-ca2-ca
13 0.000   0.000   0.000   0.000 # hc-ct3-ca2-ca
14 0.000   7.000   0.000   0.000 # ca-ca-cp-ca
15 0.000   7.000   0.000   0.000 # cp-cp-ca-ca
16 0.000   7.250   0.000   0.000 # ca-cp-cp-ca
17 0.000   2.170   0.000   0.000 # cs-cs-cs-ca
18 0.000   2.170   0.000   0.000 # ca-cs-cs-s1
19 0.000   7.250   0.000   0.000 # ca-cw-cs-ca
20 0.000   7.250   0.000   0.000 # s1-cw-cs-ca
21 0.000   7.250   0.000   0.000 # ca-cw-s1-cs
22 0.000   7.250   0.000   0.000 # cp-ca1-ca2-ca

23 0.000	7.250	0.000	0.000	# ct3-ca2-ca1-cp
24 0.000	7.250	0.000	0.000	# ha-ca-ca-cp
25 0.000	2.170	0.000	0.000	# cp-ca-cs-cs
26 0.000	7.250	0.000	0.000	# cw-cs-ca-cp
27 0.000	7.000	0.000	0.000	# cp-ca-cw-cs
28 0.000	7.000	0.000	0.000	# cp-ca-cw-s1
29 0.000	7.000	0.000	0.000	# ca-cp-ca-cs
30 0.000	7.000	0.000	0.000	# cp-cp-ca-cs
31 0.000	2.170	0.000	0.000	# cs-cs-cs-cs
32 0.000	2.170	0.000	0.000	# cw-cs-cs-cs
33 0.000	2.170	0.000	0.000	# s1-cs-cs-cs
34 0.000	7.250	0.000	0.000	# ca-cw-cs-cs
35 0.000	7.250	0.000	0.000	# s1-cw-cs-cs
36 0.000	2.170	0.000	0.000	# cw-s1-cs-cs
37 0.000	7.250	0.000	0.000	# cs-s1-cw-cs
38 0.000	7.250	0.000	0.000	# ct3-ca2-ca-ca
39 0.000	7.250	0.000	0.000	# ha-ca-ca2-ct3
40 1.300	-0.050	0.200	0.000	# ct-ct-ct4-ct3
41 0.000	0.000	0.300	0.000	# hc-ct-ct4-ct3
42 1.300	-0.050	0.200	0.000	# ct-ct-ct-ct4
43 0.000	0.000	0.300	0.000	# hc-ct-ct-ct4
44 1.300	-0.050	0.200	0.000	# ct-ct-ct-ct
45 0.000	0.000	0.300	0.000	# hc-ct-ct-ct
46 0.000	7.000	0.000	0.000	# cw-ca-cp-ca1
47 0.000	7.000	0.000	0.000	# cw-ca-cp-cp
48 0.000	2.170	0.000	0.000	# cw-cs-cs-s1
49 0.000	7.250	0.000	0.000	# ha-ca1-ca2-ca
50 0.000	7.250	0.000	0.000	# ha-ca1-ca2-ct3
51 0.000	7.250	0.000	0.000	# ha-ca1-cp-ca
52 0.000	7.250	0.000	0.000	# ha-ca1-cp-cp
53 0.000	7.250	0.000	0.000	# ha-ca-ca-ha
54 0.000	7.250	0.000	0.000	# ha-ca-cp-ca1
55 0.000	7.250	0.000	0.000	# ha-ca-cp-ca
56 0.000	7.250	0.000	0.000	# ha-ca-cp-cp
57 0.000	7.250	0.000	0.000	# ha-ca-cs-cs
58 0.000	7.250	0.000	0.000	# ha-ca-cs-cw
59 0.000	7.250	0.000	0.000	# ha-ca-cw-cs
60 0.000	7.250	0.000	0.000	# ha-ca-cw-s1
61 0.000	0.000	0.300	0.000	# hc-ct3-ct4-ct
62 0.000	0.000	0.300	0.000	# hc-ct4-ct3-hc
63 0.000	0.000	0.300	0.000	# hc-ct4-ct-ct
64 0.000	0.000	0.300	0.000	# hc-ct-ct4-hc
65 0.000	0.000	0.300	0.000	# hc-ct-ct-hc
66 0.000	7.250	0.000	0.000	# s1-cs-cs-s1

Improper Coefficients

1 2.50000	-1	2	# ca1-ca2-cp-ha
2 2.50000	-1	2	# ca2-ca1-ca-ct3
3 2.50000	-1	2	# ca-ca2-ca-ha

```
 4 2.50000  -1   2 # ca-ca-cp-ha
 5 2.50000  -1   2 # ca-cp-cs-ha
 6 2.50000  -1   2 # ca-cp-cw-ha
 7 2.50000  -1   2 # cp-ca1-ca-cp
 8 2.50000  -1   2 # cp-ca-ca-cp
 9 2.50000  -1   2 # cs-ca-cs-cw
10 2.50000  -1   2 # cs-cs-cs-s1
11 0.00000  -1   2 # ct3-ca2-ct4-hc
12 0.00000  -1   2 # ct3-ca2-hc-hc
13 0.00000  -1   2 # ct3-ct4-hc-hc
14 0.00000  -1   2 # ct4-ct3-ct-hc
15 0.00000  -1   2 # ct4-ct3-hc-hc
16 0.00000  -1   2 # ct4-ct-hc-hc
17 0.00000  -1   2 # ct-ct4-ct-hc
18 0.00000  -1   2 # ct-ct4-hc-hc
19 0.00000  -1   2 # ct-ct-ct-hc
20 0.00000  -1   2 # ct-ct-hc-hc
21 0.00000  -1   2 # ct-hc-hc-hc
22 2.50000  -1   2 # cw-ca-cs-s
```

S3. Approach to Equilibrium Molecular Dynamics (AEMD) method

In order to calculate the thermal conductivities, we rely on the AEMD method which has been fruitfully employed to investigate the thermal properties of a variety of organic and inorganic materials (see e.g. Refs. [4–6]).

In contrast to the most commonly reported methods in the literature, AEMD does not require long simulation times, as it is the case for instance in the Non Equilibrium Molecular Dynamics (NEMD) method to reach a non-equilibrium steady-state[7,8] or to achieve the numerical convergence of auto-correlation functions, as needed in the Equilibrium Molecular Dynamics (EMD) method based on the Green-Kubo formalism[9]. Additionally, the validity of the EMD and NEMD approaches for the prediction of thermal transport properties has been recently challenged[10,11]. Unlike AEMD, these two techniques rely on the calculation of an instantaneous heat flux, which has been proven to be erroneous for the many-body potentials implemented in the common version of the LAMMPS code[12].

In the AEMD approach, the thermal gradient ΔT is dissipated during a transient regime, allowing for the study of systems containing several hundred thousands of atoms under reasonable simulation times (up to ~ 3 ns). As can be seen on **Figure S2**, the fitted function (black curve) well describes the monitored time-decaying temperature offset ΔT for a 3X34X3 C8-DNTT-C8 supercell. This is of crucial interest because the prediction of the lattice thermal conductivity is strongly influenced by the size of the simulation box due to a limitation in the overall phonon mean free path owing to the reduced cell dimension. One possible way to overcome this issue is to calculate the lattice thermal conductivity for a collection of supercells of increasing size and to extract the actual bulk value from a linear regression of these data[13]. This strategy relies on the Matthiessen's rule[14], which states that all scattering mechanisms in the system behave independently. Consequently, this linear regression accounts for a transition from a ballistic to a purely diffusive regime in the infinite size limit[14].

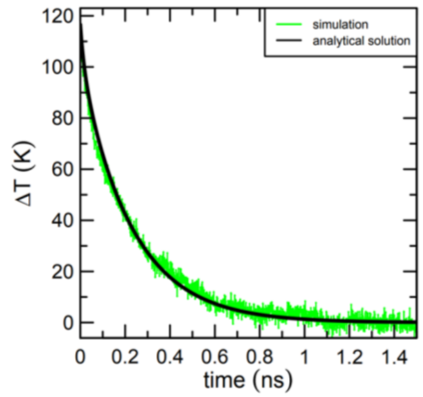

Figure S2. Time evolution of the average ΔT along the b direction of a C8-DNTT-C8 supercell; the smooth line (black) represents the result of the fitting on simulated data (noisy green line). The corresponding simulation box actually contains ~ 50 000 atoms.

As already mentioned in the core paper, we only focus in the calculations on the phononic contribution to the thermal transport in single crystals made of DNTT and C8-DNTT-C8, thus neglecting the contribution from electrons. To further address this issue, it is useful to consider that the electronic component to heat diffusion is often estimated by means of the Wiedemann-Franz law[15] $\kappa_e = L_0 T\sigma$, where $L_0 = \frac{\pi^2}{3}\left(\frac{k_b}{e}\right)^2$ [W Ω K^{-2}] is the Lorenz number. However, while this law applies to most metallic compounds (i.e., ideal gas model), it can no longer be used for molecular systems[16]. Craven and Nitzan[17] have recently proposed an alternative model (called the "Molecular Wiedemann-Franz" law), where heat conduction in OSCs is taken to be dominated by a pure electron hopping process:

$$\kappa_e = L_M T_M \sigma$$

where $L_M = \left(\frac{k_b}{e}\right)^2$ is the molecular Lorenz number and $T_M = \frac{\lambda}{k_B}$ is an effective temperature governed by the reorganization energy λ. If we assume that the electrical conductivity σ and the nuclear reorganization energy λ are typically less than 10^{-4} S m^{-1} and 10^{-1} eV in undoped films of small molecule semiconductors[18–20], the electronic thermal conductivity κ_e is

expected to be around ~ 10^{-10} W m^{-1} K^{-1}; such a value is clearly negligible in comparison to the calculated phononic contribution.

S3. Grazing-Incidence Wide-Angle X-ray Scattering (GIWAXS)

<table>
<tr><th colspan="2">DNTT</th><th colspan="2">C8-DNTT-C8</th></tr>
<tr><td>115.3 nm</td><td>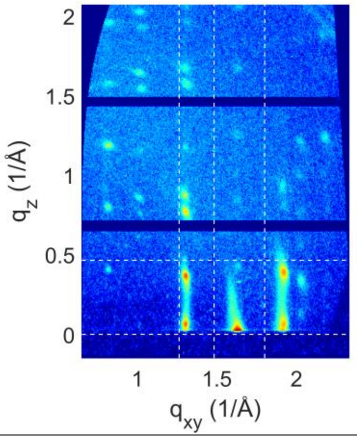
</td><td>113.3 nm</td><td>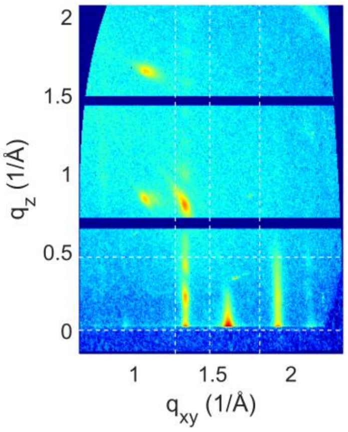
</td></tr>
<tr><td>118.1 nm</td><td>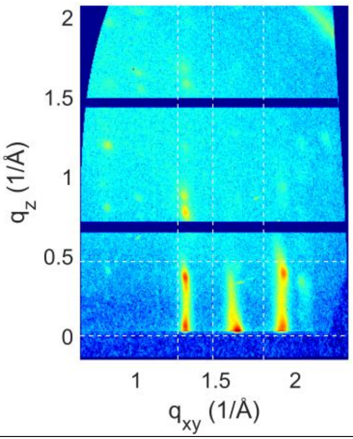
</td><td>218.8 nm</td><td>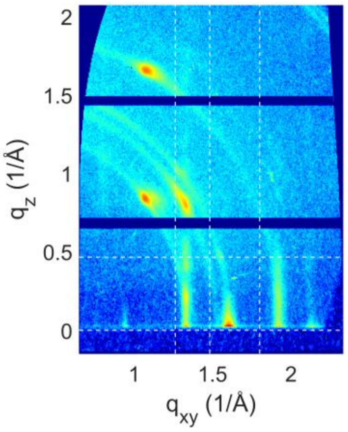
</td></tr>
<tr><td>176.1 nm</td><td>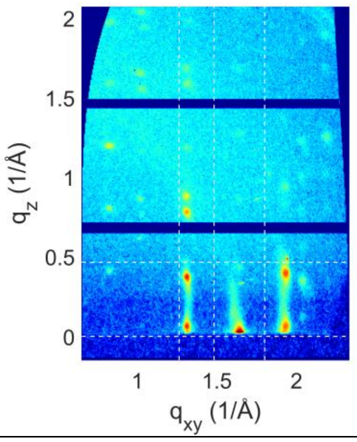
</td><td>366.5 nm</td><td>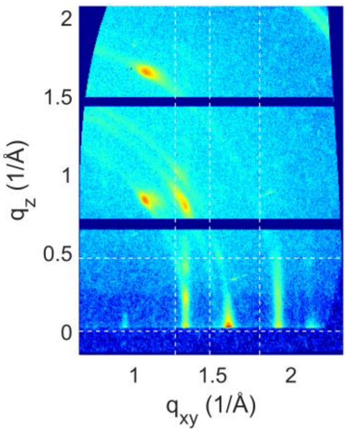
</td></tr>
</table>

237.4 nm	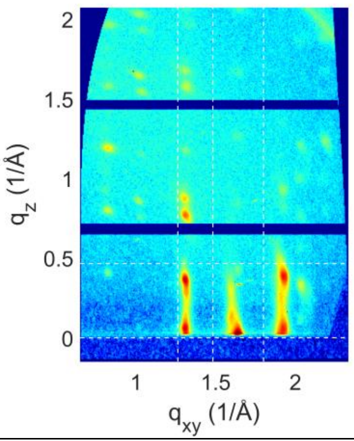	531.8 nm	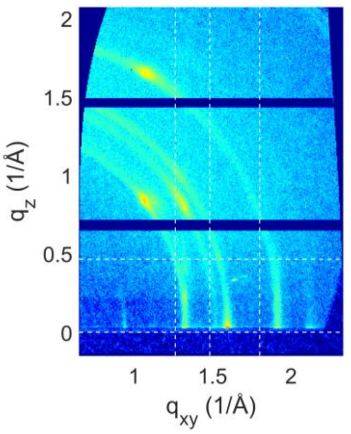

Figure S3. GIWAXS patterns of DNTT and C8-DNTT-C8 films of various thicknesses.

	DNTT				C8-DNTT-C8			
Film thickness (nm)	115.3	118.1	176.1	237.4	113.3	218.8	366.5	531.8
Average crystallite size (nm)	8.94 +-0.9	7.56 +-0.7	9.26 +-2.2	7.20 +-1.4	9.63 +- 1	7.81 +- 0.3	9.92 +-1.3	9.64 +-0.7

Table S1. Crystallite size σ determined from the Scherrer formula ($\sigma = \frac{2\pi}{FWHM}$) for the three main in-plane reflections (110), (020), (120) and averaged over the three values for DNTT and C8-DNTT-C8 films of various thicknesses.

S4. Atomic Force Microscopy (AFM)

DNTT		C8-DNTT-C8	
115.3 nm	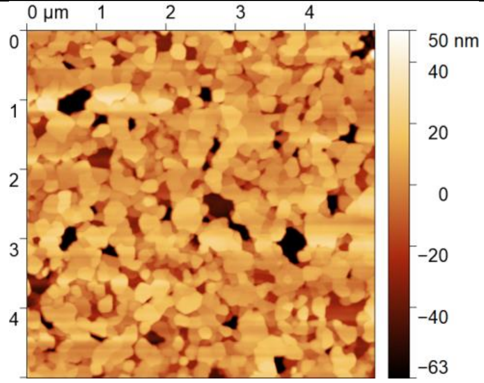 Sq = 17.85 nm	113.3 nm	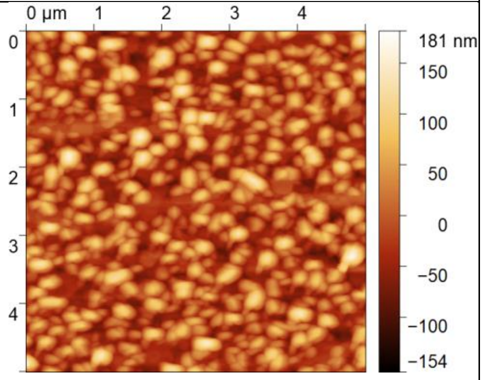 Sq = 44.34 nm
118.1 nm	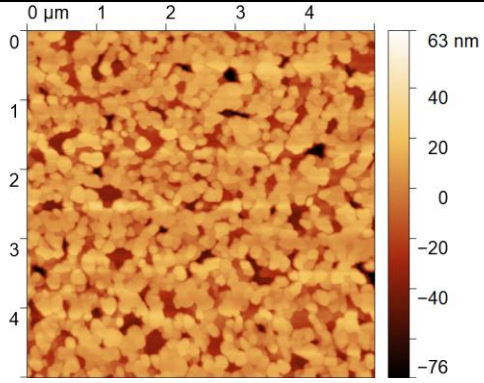 Sq = 14.97 nm	218.8 nm	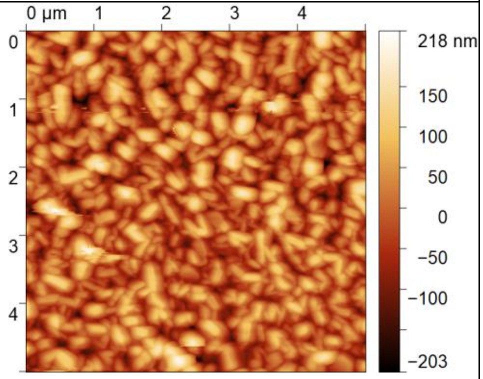 Sq = 54.32 nm
176.1 nm	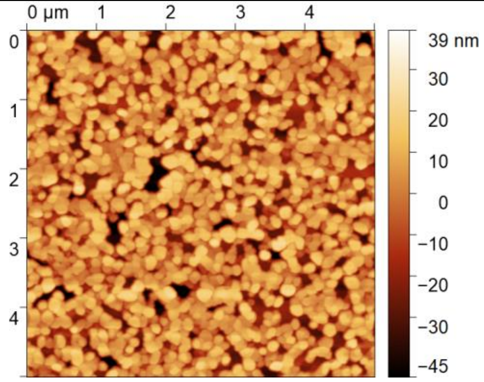 Sq = 13.05 nm	366.5 nm	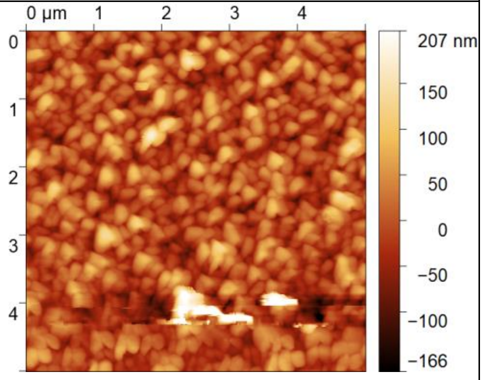 Sq = 42.28 nm
237.4 nm	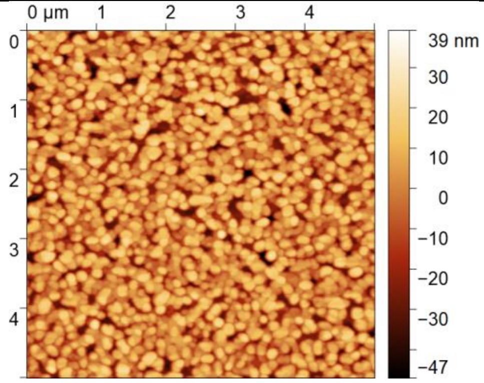 Sq = 11.99 nm	531.8 nm	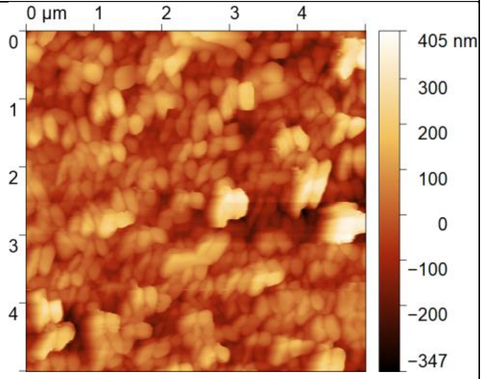 Sq = 94.37 nm

Figure S4. AFM topography images and corresponding RMS roughness (Sq) of DNTT and C8-DNTT-C8 films of various thicknesses.

S5. Thermal conductivity measurements

DNTT		C8-DNTT-C8	
115.3 nm	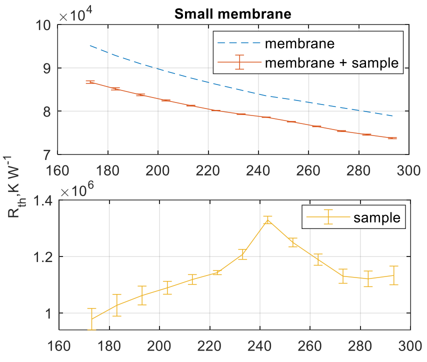	113.3 nm	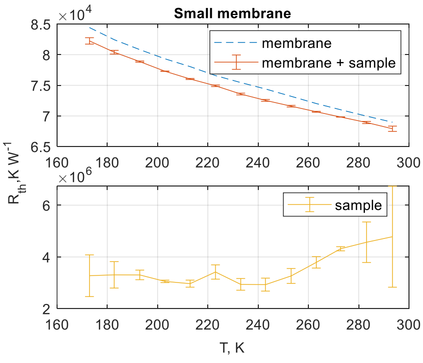
118.1 nm	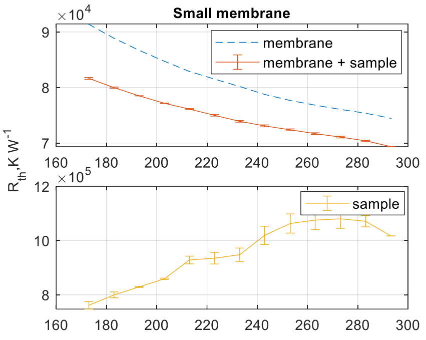	218.8 nm	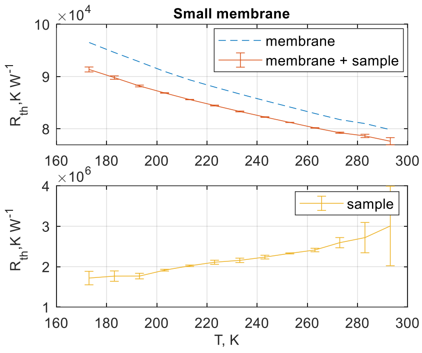
176.1 nm	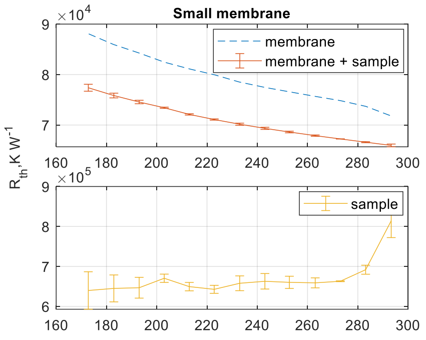	366.5 nm	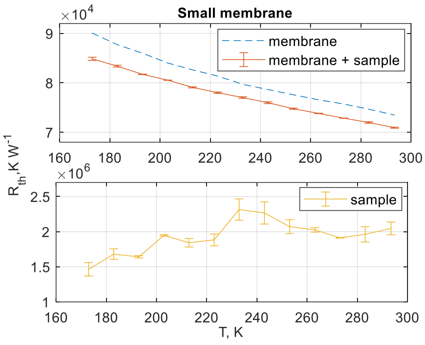
237.4 nm	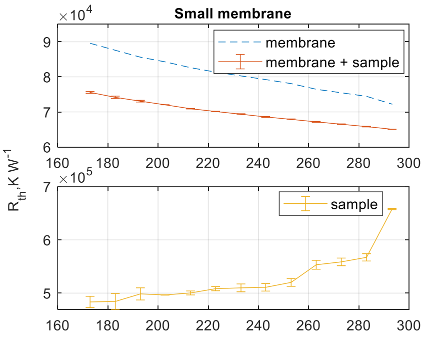	531.8 nm	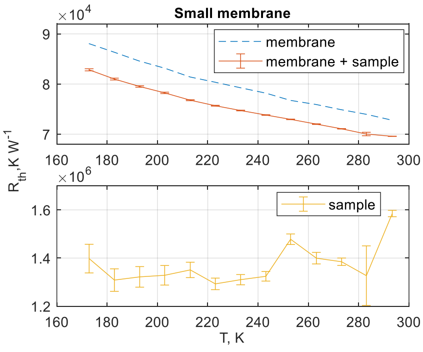

Figure S5. Measured thermal resistance of empty membranes and membranes with samples (top subplots) and derived thermal resistance of the samples (bottom subplots) for DNTT and C8-DNTT-C8 films of various thicknesses.

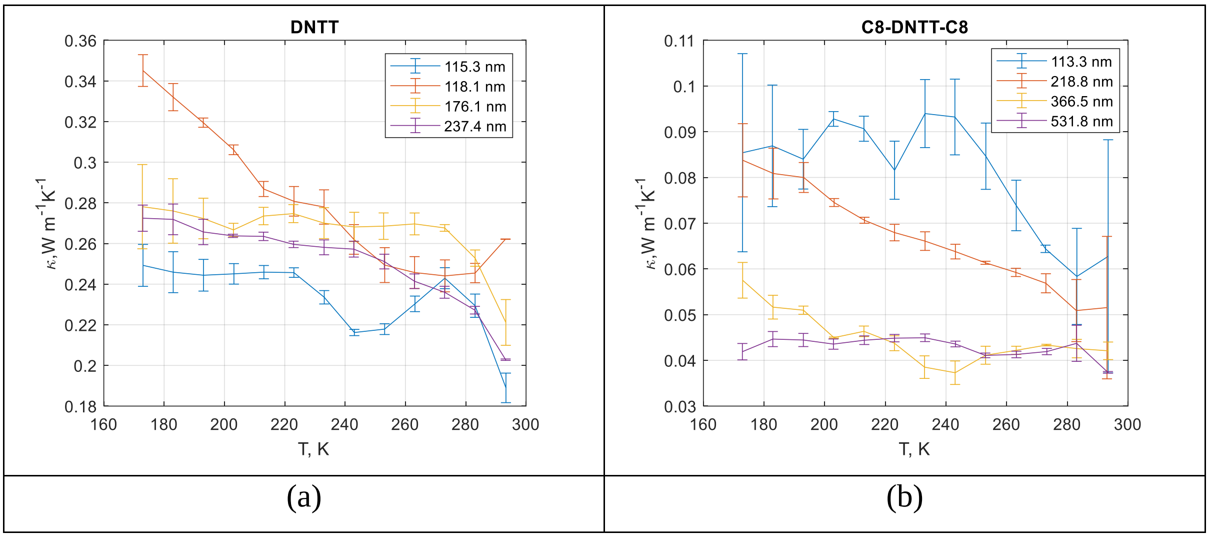

(a) (b)

Figure S6. Measured in-plane thermal conductivities of DNTT (a) and C8-DNTT-C8 (b) films of various thicknesses as a function of temperature. The error bars represent uncertainty associated with repeatability of the measurement.

References

[1] V. Linseis, F. Völklein, H. Reith, P. Woias, K. Nielsch, *J. Electron. Mater.* **2018**, *47*, 3203.

[2] D. G. Cahill, R. O. Pohl, *Phys. Rev. B* **1987**, *35*, 4067.

[3] H. Wang, W. D. Porter, H. Böttner, J. König, L. Chen, S. Bai, T. M. Tritt, A. Mayolet, J. Senawiratne, C. Smith, F. Harris, P. Gilbert, J. Sharp, J. Lo, H. Kleinke, L. Kiss, *J. Electron. Mater.* **2013**, *42*, 1073.

[4] C. Melis, R. Dettori, S. Vandermeulen, L. Colombo, D. Fisica, *Eur. Phys. J. B* **2014**, *87*, 96.

[5] M. Gueye, A. Vercouter, R. Jouclas, D. Guerin, V. Lemaur, G. Schweicher, S. Lenfant, A. Antidormi, Y. Geerts, C. MELIS, J. Cornil, D. Vuillaume, *Nanoscale* **2021**, DOI 10.1039/D0NR08619C.

[6] A. Cappai, A. Antidormi, A. Bosin, D. Narducci, L. Colombo, C. Melis, *Phys. Rev. Mater.* **2020**, *4*, 035401.

[7] F. Müller-Plathe, *J. Chem. Phys.* **1997**, *106*, 6082.

[8] D. J. Evans, G. P. Morriss, *Statistical Mechanics of Nonequilibrium Liquids*, ANU Press, Acton, Australia, **2007**.

[9] R. Kubo, M. Toda, N. Hashitsume, *Statistical Physics II*, Springer, Berlin, **1985**.

[10] P. Boone, H. Babaei, C. E. Wilmer, *J. Chem. Theory Comput.* **2019**, *15*, 5579.

[11] D. Surblys, H. Matsubara, G. Kikugawa, T. Ohara, *Phys. Rev. E* **2019**, *99*, 51301.

[12] S. Plimpton, *J. Comput. Phys.* **1995**, *117*, 1.

[13] P. K. Schelling, S. R. Phillpot, P. Keblinski, *Phys. Rev. B - Condens. Matter Mater. Phys.* **2002**, *65*, 144306.

[14] G. Fugallo, L. Colombo, *Phys. Scr.* **2018**, *93*, 043002.

[15] B. Russ, A. Glaudell, J. J. Urban, M. L. Chabinyc, *Nat. Rev. Mater.* **2016**, *1*, 1.

[16] N. Lu, L. Li, N. Gao, M. Liu, *J. Appl. Phys.* **2016**, *120*, 195108.

[17] G. T. Craven, A. Nitzan, *Nano Lett.* **2020**, *20*, 989.

[18] R. S. Sa, S. Atahan, J. Schrier, **2010**, 2334.

[19] Y. Tsutsui, G. Schweicher, B. Chattopadhyay, T. Sakurai, J. B. Arlin, C. Ruzié, A. Aliev, A. Ciesielski, S. Colella, A. R. Kennedy, V. Lemaur, Y. Olivier, R. Hadji, L. Sanguinet, F. Castet, S. Osella, D. Dudenko, D. Beljonne, J. Cornil, P. Samorì, S. Seki, Y. H. Geerts, *Adv. Mater.* **2016**, *28*, 7106.

[20] Y. Chen, Y. Zhao, Z. Liang, *Energy Environ. Sci.* **2015**, *8*, 401.
